# The British Journals: Pathology, Practical Medicine, and Therapeutics

**Published:** 1839-01

**Authors:** 


### PATHOLOGY, PRACTICAL MEDICINE, AND THERAPEUTICS.

### *Case illustrating the Origin of the Optic Nerves.* By G. Kennion, M.D.

[The following case is interesting, because, as Dr. K. observes, although the connexion of the optic nerves with the corpora quadrigemina is generally admitted, pathological facts illustrating this are not abundant.]

A gentleman, æt. sixty-six, had been gradually getting more and more blind, and when first seen by Dr. K. the symptoms were as follows:—There was complete amaurosis, with dilated and insensible pupil on the right side, and very indistinct vision (almost amounting to amaurosis) in the left eye. There was much dulness, and at the same time anxiety, in his countenance; his mind was much confused; his answers sometimes rambling: and his utterance slow and heavy. His hearing, and all his other senses, were perfect; he had the full use of all his limbs, and he never had a fit of any kind. There was no tenderness on pressing the scalp in the region affected; the pain was not influenced by any variety in his position (as erect, or horizontal), but the least quantity of wine or beer rendered it insupportable; pulse seventy-six, small and feeble; action of the heart natural; appetite tolerable; tongue white, and somewhat furred; he complained very much of a bitter taste in his mouth; the bowels were in general regular. Occasionally there was some irritability about the neck of the bladder. He stated that he had never had any fall or blow on the head.

The patient died within two months from this time, having had several fits of an epileptic character, during the ten days preceding his death.

*Sectio Cadaveris, forty-two hours after death.* The calvarium was with much difficulty removed, on account of its firm adhesion to the dura mater, the vessels of which were in a state of considerable congestion; and on the external surface of this membrane, in the occipital region, there was an effusion of about four ounces of blood. On slicing the right hemisphere of the brain from above downwards, the anterior part of the roof of the lateral ventricle appeared healthy, but the posterior portion was much softened, though not discoloured. The same was found on the left side, but to a greater degree. Behind the third ventricle, and pressing on the corpora quadrigemina, and also partially on the cerebellum, there was a tumour the size of a walnut, of a cartilaginous nature, but which in some parts was soft and easily broken up. It was partly surrounded by a softish substance, having some points of bloody infiltration. This extended for some distance into the left hemisphere, on which side there was also about ℥jss. of an amber-coloured gelatinous effusion lying over the choroid plexus, and in the posterior cornea of the lateral ventricle.

The other cavities of the body were not allowed to be examined.

*Med. Gazette. September,* 1838.

---

### *Analytical Essay on Irregular and Aggravated forms of Hysteria.* By Thomas Laycock, House-Surgeon to the York County Hospital.

[This is the continuation but not the conclusion of former papers on hysteria, and is marked by the same elaborate research and ingenuity. We refer to it chiefly with the view of recommending it to the notice of our readers. It is not susceptible of abridgment. We quote a single passage respecting the vicarious discharges of urine.]

If we examine into the question of erratic urinary discharge as a matter of fact, the proof that it has occurred is rendered as complete as possible by the following table of recorded instances.

| | Vomit. | Stool. | Ears. | Eyes. | Saliva. | Nose. | Mammæ. | Navel. | Skin. | Total. |
|---|---|---|---|---|---|---|---|---|---|---|
| In cases in the selection, | 19 | 2 | 2 | 1 | 0 | 2 | 3 | 5 | 5 | 39 |
| In cases from authors, . | 14 | 18 | 2 | 3 | 5 | 1 | 1 | 29 | 12 | 85 |
| Total . . | 33 | 20 | 4 | 4 | 5 | 3 | 4 | 34 | 17 | 124 |

Cases are omitted under the head "stool" in which there was a known communication between the rectum and bladder; in two of the number given it was ascertained that no such communication existed. The numbers in which the discharge took place from the umbilicus are not very precise. But if we suppose, that in the whole of the cases under the heads of stool and navel, there was a direct communication with the bladder, ureters, or kidneys, we have still seventy instances to account for; were they all feigned?

Haller thought that almost all secretions may, under the influence of disease, be formed by each and every secreting organ, an opinion which I think will be found to be consistent with facts. *Edin. Journ. Oct.* 1, 1838.

---

*Some Remarks on the Hooping-Cough, communicated in a Letter to Dr. R. J. Graves.* By Dr. H. C. LOMBARD, of Geneva.

[THIS is a valuable paper in relation to the statistics, pathology, and treatment of hooping-cough. Our limits will only permit us to extract the portion of it that refers to the use of carbonate of iron in this disease. We may state that since the recommendation of this remedy by Dr. Steymann we have ourselves employed it in a few cases with much benefit, and have received the same account of its effects in the hands of some of our friends.]

I come now to my specific, or rather to the remedy advised by Dr. Steymann; as the best anti-spasmodic in hooping-cough. Dr. Steymann had advised to give from four to ten grains of subcarbonate of iron in the twenty-four hours; he gave as a rule to increase one grain for each year, so that a child six years old was to take six grains in the day; but from the beginning I found that dose quite inadequate, and I increased it to twenty-four, and even thirty-six grains in young children. I have given it either with water and syrup or mixed with a cough mixture. It has never produced any inconvenience, on the contrary, I have found that all the children treated after this method were much less weakened, and recovered faster than with all other remedies. The proofs of the advantageous effects of the subcarbonate of iron have been so numerous that I can scarcely enter into the detail; however, I may give a few facts to corroborate my assertion. In a child, four years old, I gave the subcarbonate of iron, and the fits which in the preceding week had been 101 in number were reduced to sixty-six in the following week. In a weak and debilitated boy, aged seven years, the powder of belladonna had proved quite useless, when I tried the powder of iron, so prompt was the effect, that in a few days the boy was quite cured; the sister of this boy was also cured with great rapidity. A young girl, aged eight years, had eight fits in the day, and after a fortnight they were reduced to two or three very mild fits of cough. A boy, aged six years, having thirty fits of convulsive cough during the day, when he began the subcarbonate of iron, after one week the daily number was reduced to twenty-one, and in a fortnight, to eleven or twelve fits, much less violent than they were before the treatment. One of our best apothecaries had tried various remedies on his children, who were labouring under a violent attack of hooping-cough, when I advised him to try the subcarbonate of iron; the result was far beyond his and my expectations, as after three days the night fits ceased entirely, and those which occurred during the day were reduced to three or four. The last case of hooping-cough which I have treated lately was of four months' duration, and every thing had proved useless, when I gave the iron powders, which in the space of a few days succeeded in making the cough less and less.

In fact, I think I may assert with security, that the subcarbonate of iron enjoys a remarkable property to make the fits less violent, to diminish their number, and after a certain number of days to cure entirely the hooping-cough. It enjoys, besides, the advantage of strengthening the little patients, and to give them the force to resist a complaint which sometimes lasts some weeks, and generally leaves the patients weak, low, and exhausted. In some of those who have taken it, I have often seen during the first days a temporary increase of the cough, but it always subsided after two or three days, and did not prevent the good effects of the medicament. The good effects obtained by the use of the iron powders are easily explained by its anti-periodic and anti-neuralgic properties, and it shows *a posteriori*, how much the hooping-cough resembles a true neuralgic, or at all events a true nervous disease.

*Dublin Journal of Medical Science. November*, 1838.

---

*On the use of Oil in Colica Pictonum.* By J. J. Bigsby, m.d., Newark.

[We extract the following case as much to exhibit the excellent sense and judgment of the physician, as to put our readers in possession of what may really be a valuable remedy in a most troublesome disease.]

The subject was nineteen years old, stout, an apprentice to a painter. It was his second attack, the first being about a year ago, at Nottingham. He was then attended by several medical men, on account of the severity and obstinacy of the disease.

The symptoms in the present attack were those commonly observed.

Large quantities of calomel, solid opium, laudanum, castor oil, several bulky clysters (the last containing Sp. Tereb. ℥ij.) were given during the twenty-four hours succeeding my first visit, but without much effect on the complaint. Some relief to the pain, however, was derived from a sinapism to the whole abdomen. Very little blood was drawn.

At this time I asked the patient what had done him good on the previous occasion. He said, a quantity of goose grease and yeast; that this procured an evacuation when all other remedies had failed. I immediately ordered him to take about four ounces of the mixture in equal proportions; and a hard motion was the speedy consequence, together with considerable subsidence of the colic. Another dose was given the next day with similar good effect, and he has gradually recovered. He had been ill more than a fortnight without advice.

Moderate salivation was established in forty-eight hours after beginning the use of calomel and opium, but seeing that ease was procured before this period, and that these medicines had, in all probability, been unavailing in the first attack, I attribute the recovery solely to the yeast and melted fat.

My inference from this case is, that olive or castor oil, in large quantities, either or both by mouth and per rectum, should be made more prominent in the treatment of this frightful, but not often fatal, disease. The oils, I understand, are employed largely in Sheffield; but little is said about them in books.

*Med. Gazette. November* 10, 1838.

---

*Contributions on Intra-uterine Pathology.* Part I. *Notices of Cases of Peritonitis in the Fœtus in Utero.* By James Y. Simpson, m.d., Lecturer on Midwifery.

[This is such a communication as might be expected from the author of the admirable essay on the Diseases of the Placenta. It is well deserving the attention of pathologists; but we must refer them to the original, as we can only give a meager outline of the subject in this place.

An account is given, in all, of twenty-four cases, nearly one half of which came under Dr. Simpson's own notice: three fourths of them were acute. These cases amply establish the fact of the fœtus being liable to peritonitis, as none were admitted as genuine examples of the disease unless there existed in the peritoneum "one or other of the organic products of inflammation."]

*The causes* of fœtal peritonitis are divided by Dr. Simpson into those "more particularly referrible to the conditions of the mother," and those "referrible to the conditions of the fœtus." Under the former head it is observed that "in some of the cases the mother had been exposed to severe labour, or fatigue and exposure to cold and moisture, or bodily injury during her gestation; in two cases there existed general ill health during the whole of that period; and in one of these the mother herself was twice attacked with peritonitis during the course of pregnancy. In two of the cases the mothers had an attack of gonorrhœa during the period of utero-gestation, along with a syphilitic eruption in the one instance, and ulcers in the other. A third confessed that she had suffered from venereal disease; and the line of life pursued by others of the number was such as certainly freely exposed them to syphilitic infection. Indeed, it appears to me highly probable, from the investigations which I have already made upon this point, that a great proportion of those children of syphilitic mothers that die in the latter months of pregnancy may yet be shown to have perished under attacks of peritoneal inflammation."

*Symptoms.* In eleven only of the cases have we any account whatever of the condition and feelings of the mother during the period of pregnancy. In four out of these eleven cases nothing seems to have occurred that was calculated to direct the particular attention of the mother to anything peculiar in the condition of the fœtus: in three the cessation, about a fortnight before delivery, of the motions of the fœtus, as felt by the mother, was the only circumstance remembered, and in one of these cases the fœtus certainly continued to live for some time after this occurrence: in another case the motions of the infant became less and less sensible during the last two weeks of gestation: and in the three remaining instances these motions, after being much and morbidly increased for two or three days, ceased entirely, and rather suddenly, at a period varying from eleven and fifteen days to upwards of three weeks before delivery. This last combination of symptoms, namely, a great but temporary increased degree of the fœtal motions, attended occasionally with spurious pains, and followed up by the sudden and final cessation of all perceptible movements on the part of the infant, may, we believe, be not unfrequently noticed in cases of acute and fatal peritonitis of the fœtus; but at the same time it must be held in recollection that this same sequence of morbid phenomena is common to peritonitis, with all those diseases of the fœtus in utero which are similarly acute and fatal in their character; and, consequently, they cannot by any means be held as diagnostic marks of peritoneal inflammation alone. . .

"When the child has been born alive, but affected with congenital peritonitis, it has sometimes, in the more chronic forms of the disease, been emaciated, but not always so; and, in the more acute cases, when any great degree of change is observed in the condition of the child in regard to its natural condition of fatness and plumpness, we shall in general be justified in ascribing it to other causes besides the peritoneal inflammation, as we know that this disease may even prove fatal without bringing down the state of the little patient in this respect. In several cases the abdomen was swelled and fluctuating at birth; sometimes even tense and tender to the touch. With the abdominal effusion a certain degree of hydrocele generally exists in the tunicæ vaginales of male infants; and in some there has also been observed a coexistent degree of dropsical swelling in other parts of the body, as in the hands, in the upper extremities and face, in particular, or in the lower extremities and beneath the skin of the whole body.

In two of the cases, the children's skin presented at birth the yellow discoloration of jaundice. In one of them that was dead-born, the liver on inspection was found to be the seat of acute inflammation and commencing purulent infiltration. In the second case the child was born alive and survived.

Again, in other instances of congenital peritonitis, none of the equivocal symptoms here alluded to have been remarked, and the cause of death has only been discovered by the post-mortem dissection."

*Duration.* We have as yet but few data on which we can rely with any great degree of certainty for fixing the general duration of attacks of peritonitis in the fœtus. We have enough, however, I believe, to show that, contrary to the surmises of some pathologists, inflammatory action may occasionally proceed with nearly as great a degree of acuteness and severity in intra-uterine life as after birth. In some of the cases of peritonitis that have been related, the plump and unemaciated condition of the fœtus after death affords very strong evidence that the fatal morbid state under which it had suffered had not been long in its duration. In others, those symptoms of increased movement and restlessness in the fœtus, which indicated the occurrence of acute disease in some part of its system, were only remarked for one, two, or three days before its death; and in one of these cases we have further corroborative evidence of the occasional very acute character of the disease in this circumstance, that the apparent exciting cause of the peritonitis was applied only two days previous to the death of the fœtus, as indicated by a sudden and total cessation in its motions subsequently to a greatly increased degree of them. In two other cases, also, the fœtal movements ceased in the course of a day or two after the supposed exciting cause of the fœtal disease had operated upon the maternal system. Besides, the inspection of the dead body in this and in other instances presented such morbid appearances as corresponded only with those left by the more acute and rapidly fatal forms of peritonitis in the adult. Again, in other cases, the state of emaciation, and hence probably of long-continued disease, combined with the particular appearances found on dissection, do show, in as unequivocal a manner, that in these instances the inflammatory action must have been of a decidedly chronic character."

[We are happy to observe that Dr. Simpson purposes continuing his admirable researches in this curious and obscure region of pathology.]

*Edinburgh Med. and Surg. Journal. October*, 1838.

